# Itaconate: A Nexus Metabolite Fueling *Leishmania* Survival Through Lipid Metabolism Modulation

**DOI:** 10.3390/microorganisms13030531

**Published:** 2025-02-27

**Authors:** Ayyoub Kihel, Hajar El Filaly, Dounia Darif, Aicha Assouab, Myriam Riyad, Imane Nait Irahal, Khadija Akarid

**Affiliations:** 1 Biochemistry, Biotechnology and Immunophysiopathology Research Team, Health and Environment Laboratory, Ain Chock Faculty of Sciences, Hassan II University of Casablanca (UH2C), Casablanca 20100, Morocco; ayyoub.kihel@gmail.com (A.K.); elfilalyhajar@gmail.com (H.E.F.); douniadarif93@gmail.com (D.D.); aassouab@yahoo.fr (A.A.); imanenaitirahal@gmail.com (I.N.I.); 2 Immunopathology of Infectious and Systemic Diseases, Laboratory of Cellular and Molecular Pathology, Faculty of Medicine and Pharmacy, Hassan II University of Casablanca (UH2C), Casablanca 20000, Morocco; myriamriyad@gmail.com

**Keywords:** *Leishmania*, itaconate, *Acod1*, *IL1b*, *Ldlr*, *Src*, *Hadh*, M1/M2 macrophages

## Abstract

Leishmaniasis, caused by the *Leishmania* parasite, is a neglected public health issue. *Leishmania* mainly infects macrophages, where metabolic reprogramming shapes their plasticity (M1/M2), affecting the host’s resistance or susceptibility to infection. The development of this infection is influenced by immune responses, with an excessive anti-inflammatory reaction linked to negative outcomes through the modulation of various mediators. Itaconate, produced by the *Acod1* gene, is recognized for its anti-inflammatory effects, but its function in leishmaniasis is not well understood. This study aimed to investigate the potential role of itaconate in leishmaniasis. Using transcriptomic data from *L. major*-infected BMDMs, we assessed the expression dynamics of *Il1b* and *Acod1* and performed pathway enrichment analysis to determine the profile of genes co-expressed with *Acod1*. Early *Acod1* upregulation followed by later *Il1b* downregulation was noted, indicating a shift towards an anti-inflammatory response. Among the genes co-expressed with *Acod1*, *Ldlr*, *Hadh*, and *Src* are closely associated with lipid metabolism and the polarization of macrophages towards the M2 phenotype, thereby creating a favorable environment for the survival of *Leishmania*. Overall, these findings suggest that *Acod1* and its co-expressed genes may affect the outcome of *Leishmania* infection by modulating host metabolism. Accordingly, targeting itaconate-associated pathways could provide a novel therapeutic strategy for leishmaniasis.

## 1. Introduction

Under inflammatory conditions, the activation of immune cells is characterized by notable metabolic changes. These changes are mediated by the upregulation of specific enzymes that are typically expressed at low levels or that remain inactive under normal conditions [1,2]. Through a variety of processes, including signal transmission, regulation of gene expression, and modification of protein activity, metabolites can impact immune cell function [3,4,5]. The study of immunometabolism, or the interplay between metabolism and the immune response, has recently increased significantly in popularity, and has revealed novel therapeutic possibilities for translational medicine.

Itaconate is an immunometabolite produced by macrophages and other myeloid cells through the enzyme cis-aconitate decarboxylase (ACOD1), also known as immunoresponsive gene 1 (IRG1), which transforms cis-aconitate, an intermediate in the Krebs cycle, into itaconate. *Acod1* expression is cell- and tissue-specific; it is expressed at very low levels under normal conditions. However, its expression is upregulated under stress conditions, particularly in response to inflammatory stimuli [6,7,8]. The structural and chemical similarity of itaconate to other metabolites, such as succinate, fumarate, malonate, and phosphoenolpyruvate, has provided insight into its antimicrobial properties. Beyond its role as an antimicrobial metabolite, itaconate has also been shown to exhibit a wide range of immunoregulatory functions [9]. Itaconate has been shown to inhibit succinate dehydrogenase (SDH) activity. Indeed, the addition of itaconate to LPS-activated macrophages impaired SDH activity, leading to decreased expression of inflammatory transcripts [10,11]. Furthermore, in Zika virus-infected mouse neurons, *Acod1*-dependent itaconate synthesis prevented the growth of the virus by inhibiting SDH activity, supporting its role as anti-inflammatory agent through a succinate-dependent mechanism [12]. Additionally, itaconate impacts glycolysis by inhibiting GAPDH. One study demonstrated that the itaconate derivative 4-octyl itaconate (4-OI) inhibits GAPDH activity, resulting in reduced aerobic glycolysis and decreased production of pro-inflammatory mediators such as IL-1β, nitric oxide synthase (NOS) 2, and TNF in activated macrophages [13]. Itaconate can regulate glycolysis by inhibiting other key metabolic enzymes, such as lactate dehydrogenase A (LDHA) and fructose-6-phosphate 2-kinase [14]. In addition, itaconate has been shown to impact type I interferon (IFN) responses. During influenza A virus infection, itaconate reduces the phosphorylated STAT1 levels in human macrophages, thereby suppressing *Cxcl10* expression and demonstrating its immunosuppressive role in regulating IFN-I signaling [15,16]. A significant role of itaconate is in the suppression of the NLRP3 inflammasome, an innate immune component that drives the secretion of proinflammatory cytokines such as IL-1β and IL-18 [17]. Indeed, exogenous itaconate reduced the release of IL-1β from macrophages without affecting its transcription, suggesting direct modulation of NLRP3 activity [18]. On the other hand, *Acod1*^−*/*−^ BMDMs stimulated with LPS and ATP produced high levels of IL-1β and IL-18; however, the exact mechanism through which itaconate inhibits inflammasome activation and IL-1β and IL-18 release remains unclear [11,19]. Overall, these studies provide valuable insights into the role of itaconate in modulating immune responses through its anti-inflammatory properties. Although its functions have not been fully explored, the evidence suggests that it plays a crucial role in the response to infections.

Leishmaniasis is a major tropical and subtropical infectious disease and is caused by the protozoan parasite *Leishmania*. Despite advancements in vaccine development, treatment, and diagnosis, many challenges persist [20,21]. During *Leishmania* infection, macrophages serve as the main cellular target, housing the parasites within parasitophorous vacuoles (PVs). Metabolic changes in *Leishmania*-infected macrophages, particularly in M1 and M2 macrophages, have been associated with resistance and susceptibility to the infection, respectively [22]. As an auxotrophic parasite with intricate nutritional requirements, *Leishmania* manipulates host metabolic pathways to produce essential metabolites for its survival [21,23]. For instance, *Leishmania* scavenges essential nutrients like arginine, which not only promotes parasite growth but also enables their survival by modulating the immune response of macrophages [24,25,26,27]. Additionally, the activation of host AMPK in *Leishmania*-infected macrophages, which is associated with a metabolic shift from glycolysis to oxidative metabolism, has been shown to be a key mechanism for parasite survival [23]. Furthermore, during the early stages of infection, *Leishmania*-infected macrophages exhibit an upregulation in the transcription of several metabolic genes, a process correlated with parasite survival [23,28].

In infectious diseases where pathogens like *Leishmania* evade the host’s pro-inflammatory mechanisms while exploiting anti-inflammatory pathways to their advantage, targeting the itaconate-mediated response could be a promising therapeutic strategy to improve pathogen clearance. Given the increasing evidence connecting macrophage metabolism to the host’s anti-parasitic responses, this study aimed to elucidate the role of itaconate in the context of leishmaniasis and uncover its potential as a therapeutic target by integrating transcriptomics and bioinformatics data, thereby presenting a highly promising approach for the treatment of the disease.

## 2. Material and Methods

### 2.1. Transcriptomic Data

The transcriptome dataset normalized matrix (quantile normalization) was obtained from Gene Expression Omnibus (GEO) dataset GSE31995 [29]. This dataset contains the transcriptomes of murine bone marrow-derived macrophages (BMDMs) infected with *L. major* promastigotes at different time points. Briefly, the transcriptome dataset was generated using GeneChip Mouse Gene 1.0 ST arrays to analyze the gene expression of BMDMs from BALB/c mice infected with *L. major*. The samples were collected from non-infected and parasite-infected macrophages at different time points post-infection. A T0 (0h) non-infected condition was used as an additional control. For each biological condition, three independent biological replicates were included. The complete dataset is publicly available at https://www.ncbi.nlm.nih.gov/geo/query/acc.cgi?acc=GSE31995 (accessed on 15 December 2024) [30].

### 2.2. Gene Expression Visualization of Acod1 and Il1b Gene Expression

The expression data for the *ACOD1* and *IL*-1β genes retrieved from the GSE31995 dataset were visualized using GraphPad Prism (v8.0.2). Heatmaps and line graphs with connecting symbols were generated to show the relative expression levels of these genes across the different experimental conditions.

### 2.3. Identification and Gene Set Enrichment Analysis of Genes Co-Expressed with Upregulated Acod1 Expression 

To identify the genes that are co-expressed with *Acod1*, a correlation analysis was performed using the “*limma*” package (Linear Model for Microarray, version 3.0.0) using R software (v4.3.3), as previously described by Ritchie and colleagues. *Acod1* expression was used as a predictor in this analysis [31].

Positively correlated genes were filtered according to a cut-off of an adjusted *p*-value < 0.05, which were used for further analysis. The significant positively correlated genes that were used as inputs were *Mmp13, Il1rn, Ripk2, Sod2, Car13, Pcdh7, Tgm2, Irak3, Dram1, Src, Itga5, Tfap2e, Sav1, Stx11, Clec4e, Rab11, Fip1, Ldlr, Mmp14, Tmem171, Pgs1, Traf1, Cd69, Tpm4, Gsap, Mcoln2*, and *Clcn7*. These genes were input into EnrichR (https://maayanlab.cloud/Enrichr/, accessed on 15 December 2024) to perform gene set enrichment analysis using the Hallmarks Molecular Signatures Database (Hallmarks MsigDB) to identify significantly enriched pathways [32,33]. Those with an adjusted *p*-value (also known as q-value) < 0.05 were considered significant. Pathways with higher EnrichR combined scores were visualized.

The significant pathway enrichment results were visualized using the “*ggplot*” package in Rstudio (v4.3.3) [34]. A bar chart was generated to display the combined score of each pathway. To further explore the relationships between the significant pathways and implicated genes, network visualization was performed using Cytoscape (v3.10.2.) [35].

### 2.4. Statistical Analysis

The statistical significance of the differences in gene expression between the non-infected and *L. major*- infected BMDMs across the different time points was analyzed using two-way ANOVA with the appropriate post hoc multiple comparisons in GraphPad Prism (v8.0.2) to assess statistical significance.

## 3. Results

### 3.1. Kinetics of Il1b and Acod1 Gene Expression Levels in L. major-Infected Bone Marrow-Derived Macrophages (BMDMs)

**Figure 1:**
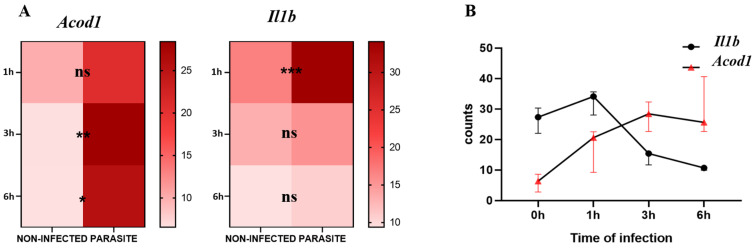
*Acod1* and *Il1b* gene expression levels from GSE31995 dataset. (**A**) Expression counts of infected and non-infected BMDMs at 0 h, 1 h, 3 h, and 6 h. (**B**) Visualization of kinetics of expression of both genes at the three timepoints. ns, not significant; *** *p*-value < 0.001; ** *p*-value < 0.01; * *p*-value ≤ 0.05.

For the analysis of the kinetics of *Il1b* and *Acod1* gene expression, we observed that *Il1b* was significantly upregulated in infected compared with non-infected BMDMs at 1 h post-infection (*p*-value = 0.0002) while *Acod1* did not show any significant variation at the same timepoint. At 3 h and 6 h post-infection, *Il1b* expression lost statistical significance, whereas *Acod1* showed significant upregulation in *L. major*-infected macrophages in comparison to non-infected BMDMs at both time points (*p*-value = 0.0077 and *p*-value = 0.0121, respectively) (Figure 1A). By visualizing the *Il1b* and *Acod1* gene expression levels on the same graph, we can observe that *Il1b* expression was prominent at an early time point (1 h), while *Acod1* was upregulated at later time points (3 h and 6 h) (Figure 1B).

### 3.2. Transcriptomics

**Figure 2:**
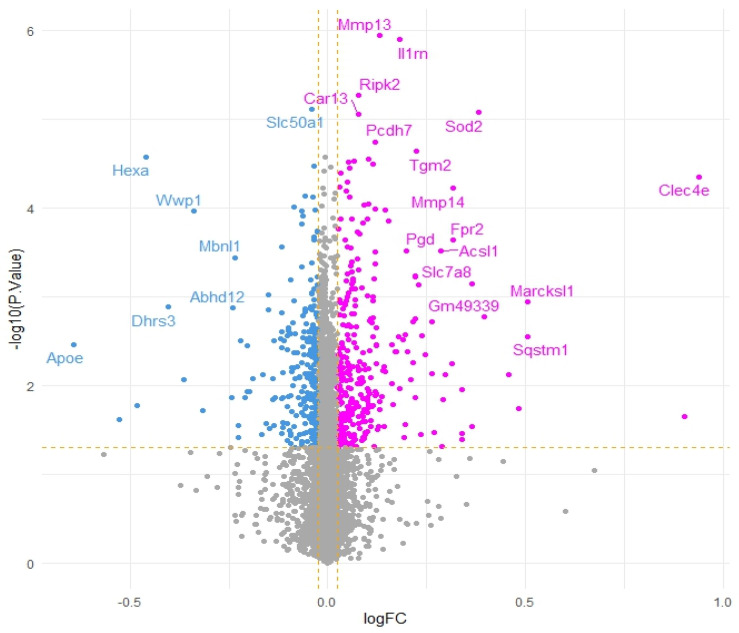
Volcano plot of genes co-expressed with *Acod1* gene expression upregulation (pink dots represent positively correlated genes, blue dots represent negatively correlated genes and grey dots indicate genes that do not show significant correlation. Yellow dashed lines represent thresholds for logFC and −log10 (*p*-value).

To further corroborate our speculations, genes correlated with *Acod1* gene expression upregulation were identified. A total of 14 genes were found to be downregulated and 27 genes were found to be upregulated, while the remaining genes showed no significant variation (Figure 2).

**Figure 3:**
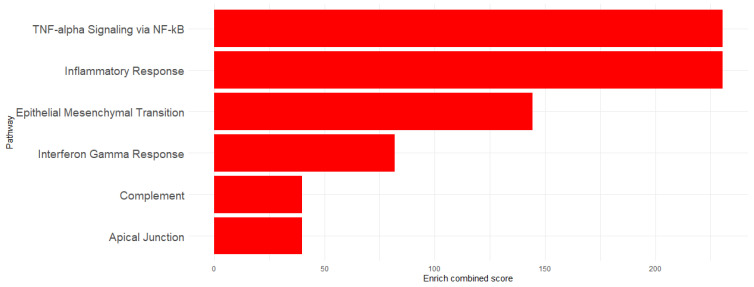
Enriched pathway visualization using EnrichR combined scores.

**Figure 4:**
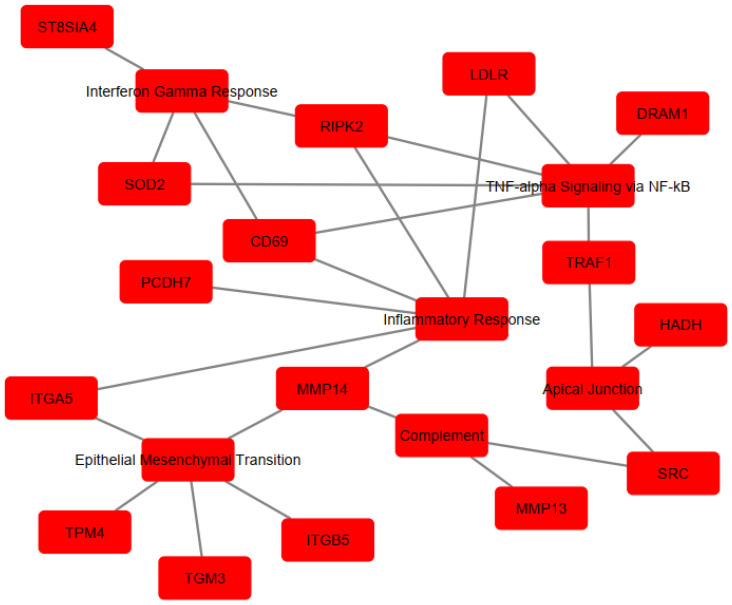
Cytoscape network visualization of enriched pathways and associate gene sets. Red nodes represent genes co-expressed with *Acod1* or their associated pathway, while edges (lines) indicate interactions or associations between them.

To determine the implication of the upregulated genes, enrichment analysis was performed using Hallmarks MSigDb. The analysis revealed significant enrichment of the following pathways: TNF-α signaling via the NF-κB pathway (EnrichR combined score = 230.6618), Inflammatory Response (EnrichR combined score = 230.6618), Epithelial–Mesenchymal Transition (EnrichR combined score = 144.2694), Interferon-γ Response (EnrichR combined score = 81.89774), Apical Junction (EnrichR combined score = 39.89133), and Complement (EnrichR combined score = 39.89133) (Figure 3). The genes associated with each pathway are detailed in Table 1 and visualized in Figure 4, which shows the network map of the key genes co-expressed with *Acod1* and their associated hallmark pathways. In this network, the inflammatory response pathway appears to be the central node, indicating that all the genes in the network are related to this biological process. Several genes in this network are directly or indirectly associated with lipid metabolism, including *Ldlr*, *Hadh*, and *Src*, suggesting that *Acod1* upregulation could reshape both metabolic and inflammatory responses during *Leishmania* infection.

The genes are involved in immune and metabolic responses. Specifically, *Cd69* and *Src* are involved in immune cell activation. *Traf1*, *Ripk2*, and *Dram1* contribute to inflammatory regulation by modulating TNF and NOD-like receptor-mediated signaling and autophagy, respectively. *Itga5*, *Itgb5*, *Tpm4*, and *Pcdh7* are associated with cell adhesion/migration, cytoskeletal dynamics, and cell–cell interactions. The *St8sia4* gene modulates immune cell interactions through sialylation. In terms of metabolic processes, three genes (*Sod2*, *Ldlr*, and *Hadh*) were implicated in regulating the oxidative stress response and lipid metabolism/cholesterol homeostasis. Other genes, including *Mmp14*, *Mmp13*, and *Tgm3*, are involved in protein cross-linking and extracellular matrix remodeling. Notably, some of these genes play dual roles, affecting “immuno-metabolism”, such as *Src*, which has been shown to induce lipid synthesis and regulate the cell’s lipid stores [36,37,38].

## 4. Discussion

Our results showed that *Acod1* and *Il1b* were expressed at different timepoints, with *IL*-1β being expressed in *L. major*-infected BMDMs at early timepoints, while *Acod1* expression occurred later in the course of the infection. Mainly secreted by macrophages, IL-1β is a potent pro-inflammatory cytokine whose production is partially mediated through NLRP3 inflammasome activation. It plays a crucial role in host defense responses to infection [39,40].

During leishmaniasis, IL-1β production triggers Inducible Nitric Oxide Synthase (iNOS) activation and Nitric Oxide (NO) production, which are responsible for parasite clearance [41]. This suggests that *Acod1* expression might mitigate the effects of *Il1b* expression, thereby mediating an anti-inflammatory response in BMDMs infected with *L. major* and creating a favorable environment for *Leishmania* survival. In line with our hypothesis, Palacios et al. demonstrated that itaconic acid abrogated the control of parasite replication in M1 activated macrophages infected with *L. infantum* [42]. In fact, macrophages are highly plastic cells that respond to their environment through polarization, which plays an essential role in the outcome of *Leishmania* infections. M1 macrophages are well known as microbicidal immune cells responsible for killing the *Leishmania* parasite through the secretion of pro-inflammatory cytokines, including Tumor Necrosis Factor-alpha (TNF-α), IL-1β, IL-6, and IFN-γ. This M1 phenotype also promotes the production of Reactive Oxygen Species (ROS) and nitrogen species such as NO [22,43,44,45]. In contrast, M2 macrophages are associated with anti-inflammatory responses. In the context of leishmaniasis, these immunosuppressive cells have been tightly linked to *Leishmania* survival through the production of IL-10 and TGF-β, while simultaneously inhibiting ROS production [43].

The differential kinetics of *Acod1* and *Il1b* expression in *L. major*-infected BMDMs may support the hypothesis that itaconate contributes to the progression of leishmaniasis by inhibiting M1 macrophage polarization and potentially driving an M2-mediated response. Few studies have demonstrated the role of itaconate in inhibiting M1 macrophages activation, while its role in M2 macrophages remains largely unknown [46]. Multiple studies have shown that M1 macrophages primarily rely on glycolysis to produce energy, unlike M2 macrophages, which depend on oxidative phosphorylation and lipid metabolism [47]. On the one hand, itaconate has been shown to inhibit glycolysis [46], which may contribute to the inhibition of M1 polarization. On the other hand, itaconate has been implicated in lipid metabolism. One study demonstrated that *Acod1*-knockout (KO) mice exhibited exacerbated hepatic lipid accumulation during sepsis, improved glucose oxidation, and reduced fatty acid oxidation. Likewise, in vitro treatment of hepatocytes (AML12 cells) with 4-octyl itaconate (4-OI)**,** a derivative of itaconate, promoted mitochondrial fatty acid uptake and clearance by upregulating the expression of oxidative phosphorylation proteins and fatty acid β-oxidation enzymes [48]. Overall, these data indicate that itaconate supports *Leishmania* survival, potentially by promoting macrophage polarization toward the M2 phenotype through modulation of lipid metabolism.

Gene enrichment analysis of the genes co-expressed with upregulated *Acod1* expression in *L. major*-infected BMDMs, we identified six significant pathways and genes implicated in immune and metabolic processes. Among these, *Ldlr*, *Hadh* and *Src* are implicated in lipid metabolism, which is tightly linked to macrophage polarization.

The *Hadh* gene encodes short-chain L-3-hydroxyacyl-CoA dehydrogenase (HADH), a crucial enzyme in fatty acid oxidation that mediates the third step of fatty acid oxidation in mitochondria [49]. There are two forms of HADH: the alpha subunit (HADHA) and the beta subunit (HADHB), both of which are integral components of the mitochondrial trifunctional protein (MTP) that facilitates multiple steps in fatty acid metabolism [36]. Due to its critical role in β-oxidation, HADH is thought to be associated with the polarization of macrophages toward the M2 phenotype. To our knowledge, no studies have investigated the involvement of the HADH or β-oxidation in leishmaniasis. However, its role has been studied in the case of *Mycobacterium tuberculosis* (*M. tuberculosis*) infections. Although distinct from the *Leishmania* parasite, both are intracellular pathogens that target macrophages as their host cells. Interestingly, Chandra and colleagues showed that the inhibition of fatty acid oxidation by chemicals, such as trimetazidine (TMZ), which blocks the 3-ketoacyl-CoA thiolase activity of HADHB, restricts the growth of *M. tuberculosis*. In addition, TMZ treatment of *M. tuberculosis*-infected BMDMs resulted in mitochondrial ROS (mROS) production, which promotes NADPH oxidase recruitment and autophagy to limit bacterial growth [50]. According to previous reports, ROS are highly produced by M1 macrophages, suggesting that HADH inhibition might control *M. tuberculosis* infection by promoting the polarization of these pro-inflammatory immune cells.

The Src proto-oncogene, also named c-Src, is a critical non-receptor tyrosine kinase encoded by the *SRC* gene in humans and contributes to cellular proliferation, differentiation, migration, adhesion, and survival [51]. Likewise, Src is indirectly linked to lipid metabolism: this protein was shown to interact with Lipin-1, an essential enzyme that operates as a phosphatidate phosphatase (PAP) to regulate lipogenesis [38]. In addition to its role in lipogenesis, Lipin-1 plays a crucial role in the oxidation of fatty acids. Schilke et al. showed that Lipin-1 modulates lipid metabolism and oxidative phosphorylation within macrophages through the catabolism of fatty acids and by enhancing β-oxidation in response to diverse pro-resolving stimuli including IL-4, palmitate (a free fatty acid), and apoptotic cellular debris [52]. IL-4 is recognized as a potent polarizing cytokine that polarizes macrophages toward the M2 phenotype. Interestingly, Chandran et al. demonstrated that Lipin-1’s transcriptional co-regulator activity is essential for IL-4-mediated macrophage polarization [53]. In addition to its interaction with Lipin-1, SRC is suspected to be directly involved in driving M2 macrophage polarization. Notably, Xiang Hu et al. observed that pretreatment of BMDMs with SRC inhibitor-1 promoted the expression of M1 macrophage markers in response to IFN-γ and suppressed the expression of M2 macrophage markers in response to IL-4 [54]. In summary, we suggest that the co-expression of *Hadh* and *Src* genes with *Acod1* in *L. major*-infected BMDMs may support macrophage polarization toward the M2 phenotype, thereby creating a favorable niche for *Leishmania* survival within the macrophage.

*Ldlr* is a cell membrane glycoprotein that functions in binding and internalizing circulating cholesterol-containing lipoprotein particles [37]. Several studies have shown that *Ldrl* is upregulated during *Leishmania* infection [30,55,56]. Semini and colleagues showed that infection of BMDMs by both *L. major* and *L. mexicana* leads to the upregulation of *Ldlr*, which was correlated with a significant increase in cholesterol levels in PVs and it was found to form a halo around the parasites. Interestingly, the host cell’s cholesterol was not only trafficked to PVs but also became incorporated into the parasite’s membrane [56]. In another study investigating peritoneal macrophages from Swiss mice infected with *L. amazonensis,* it was demonstrated that intracellular amastigotes cultured in a cholesterol-free medium were more sensitive to ketoconazole and miconazole (drugs inhibiting ergosterol biosynthesis by *Leishmania* parasites), suggesting that *Leishmania* parasites can use cholesterol to replace ergosterol to maintain their membrane properties [57]. Parihar et al. showed that inhibition of cholesterol biosynthesis by simvastatin (HMG-CoA reductase inhibitor) reduced the growth of the *L. major* amastigote form in primary-culture macrophages. The topical application of simvastatin relieved ear and footpath swelling and ulceration and reduced the parasite burden in both *BALB/c* and *C57BL/6* mice models infected with *L. major* [58]. These findings suggest that host cholesterol may be used in the construction of the *Leishmania* parasite’s membrane, affecting its virulence and pathogenicity. In brief, the co-expression of *Ldlr* with *Acod1* in *L. major*-infected BMDMs suggest that cholesterol is actively internalized by infected macrophages to ensure the survival and proliferation of the parasite within.

Overall, the *Acod1* gene, which encodes aconitate decarboxylase, which is responsible for itaconate production, could regulate inflammation by promoting M2 macrophage polarization and inhibiting M1 macrophage polarization. This inhibition may promote *Leishmania* survival and multiplication within macrophages. Additionally, *Src* and *Hadh*, which are co-expressed with *Acod1*, are likely involved in lipid metabolism processes such as β-oxidation, a characteristic of the M2 macrophage phenotype that may further support *Leishmania* survival. Likewise, *Ldlr* also plays a role in the pathogenesis and persistence of *Leishmania* by ensuring cholesterol uptake.

Although these findings are compelling and have potential, it is crucial to note that our conclusions are based on a bioinformatics analysis. Experimental validation is necessary to confirm the role of itaconate in the context of leishmaniasis.

Overall, these findings suggest that itaconate promotes a favorable intracellular environment for *Leishmania* survival, both directly and through the co-expression of genes during infection. This highlights itaconate as a potential therapeutic target for treating leishmaniasis.

## Figures and Tables

**Table 1:** EnrichR results using Hallmark Msig_Database.

Pathway	Overlap	*p*-Value	Adjusted *p*-Value	odd_R	Combined Score	Genes
TNF-alpha Signaling via NF-kB	6/200	2.68 × 10^−6^	4.16 × 10^−5^	17.97999	230.6618	*Dram1* ***/*** *Ripk2* ***/*** *Traf1* ***/*** *Cd69* ***/*** *Sod2* ***/*** *Ldlr*
Inflammatory Response	6/200	2.68 × 10^−6^	4.16 × 10^−5^	17.97999	230.6618	*Mmp14* ***/*** *Ripk2* ***/*** *Pcdh7* ***/*** *Itga5/Cd69* ***/*** *Ldlr*
Epithelial Mesenchymal Transition	5/200	4.71 × 10^−5^	4.87 × 10^−4^	14.47985	144.2694	*Mmp14* ***/*** *Itgb5* ***/*** *Tpm4* ***/*** *Itga5* ***/*** *Tgm2*
Interferon Gamma Response	4/200	6.69 × 10^−4^	0.005185	11.20408	81.89774	*Ripk2* ***/*** *St8sia4* ***/*** *Cd69* ***/*** *Sod2*
Apical Junction	3/200	0.007415	0.038311	8.134038	39.89133	*Src* ***/*** *Traf1* ***/*** *Hadh*
Complement	3/200	0.007415	0.038311	8.134038	39.89133	*Mmp14* ***/*** *Mmp13* ***/*** *Src*

## Data Availability

The datasets used in this study are available online (GEO dataset, https://www.ncbi.nlm.nih.gov/geo/query/acc.cgi?acc=GSE31995, Date of access: 15 December 2024).
